# Mechanistic insights into combined prefrontal tDCS and vagal nerve stimulation for stress regulation

**DOI:** 10.3389/fpsyg.2026.1872276

**Published:** 2026-07-14

**Authors:** Sumit Roy, Marie-Anne Vanderhasselt

**Affiliations:** 1 Department of Medical Psychology and Medical Sociology, Center of Medical Psychology and Translational Neuroscience, Medical Faculty, Ruhr University Bochum, Bochum, Germany; 2 Ghent Experimental Psychiatry (GHEP) Lab, Faculty of Medicine and Health Sciences, Department of Head and Skin, Ghent University, Ghent, Belgium

**Keywords:** emotional regulation, NIBS (non-invasive brain stimulation), stress regulation, vagus nerve, taVNS, tDCS

## Abstract

Stress disrupts prefrontal cortex (PFC) functioning through converging top-down and bottom-up mechanisms, posing a challenge for interventions aimed at restoring adaptive regulation under stress. In this context, this conceptual review explores the mechanistic rationale for combining transcranial direct current stimulation (tDCS) and transauricular vagus nerve stimulation (taVNS) as a dual-modality approach to stress regulation. We first outline the neurobiological substrates of the stress response, focusing on prefrontal dysregulation mediated by the locus coeruleus–norepinephrine (LC-NE) system. We then review the current evidence for tDCS and taVNS as individual tools for modulating stress-related behavioral, physiological, and subjective outcomes. Finally, we develop a mechanistic rationale at multiple levels (cellular, systems, and network) for their combined application, grounded in the state-dependency and priming effects of tDCS. While tDCS modulates top-down cortical excitability via targeted prefrontal stimulation, taVNS activates bottom-up vagal afferents, dampening autonomic hyperarousal and reducing LC-NE activity, as inferred from indirect physiological measures. We propose that taVNS-mediated reduction in autonomic hyperarousal may contribute to a more favorable neural context for tDCS-induced neuroplasticity, with the two techniques engaging complementary regulatory loops that may be difficult to engage simultaneously with either technique alone. Combined tDCS and taVNS represents a theoretically motivated framework with potential for clinical translation, pending systematic empirical validation. Key challenges for the combination include optimizing stimulation timing, sequencing, dosage, and addressing inter-individual variability. Future work should systematically examine synergistic and antagonistic interaction effects and explore clinical translation in populations characterized by prefrontal hypoactivity and autonomic dysregulation.

## 1 Introduction

Stress is a widespread issue affecting millions globally (Magomedova and Fatima, 2025), with prevalence continuing to rise, especially in younger generations (Piao et al., 2024). Stress triggers and worsens mental disorders, making it a major risk factor for poor health (Candeias et al., 2024). It acts as a transdiagnostic process, increasing vulnerability to depression, anxiety, and related disorders (Eberle and Maercker, 2023). The economic burden is significant, costing tens of billions annually through absenteeism, reduced productivity, and treatment (Hassard et al., 2018). Current treatments are limited in their ability to regulate stress responses directly, underscoring the need for minimally invasive, easy, and effective interventions. Stress regulation, therefore, remains a promising target for prevention and treatment for a range of mental disorders.

Recently, one promising non-pharmacological approach for reducing stress has gained considerable attention: non-invasive brain stimulation (NIBS) techniques (Nunes Sá et al., 2024). These methods alter neural activity without surgical procedures, offering a safe and scalable option. Among the available NIBS techniques, tDCS and taVNS stand out for their ease of use, affordability, and promising effects on stress-related neurophysiological and behavioral outcomes (Davidson et al., 2024; Hart-Pomerantz et al., 2024; Bremner et al., 2020). Importantly, these two approaches engage mechanistically distinct yet functionally complementary pathways: tDCS modulates top-down cortical excitability through targeted electrical fields (Feeser et al., 2014; Roy et al., 2025), while taVNS activates peripheral vagal afferents, affecting bottom-up brainstem and limbic circuitry (Molaeizadeh et al., 2026; Hilz, 2022). Additionally, tDCS effects on the PFC are known to be state-dependent (Allaert et al., 2026; Esposito et al., 2022), with hyperarousal disrupting its effects (Aksu et al., 2022; Frase et al., 2019; de Lima et al., 2019; Miler et al., 2026). While taVNS, by reducing autonomic hyperarousal via ascending vagal afferents, help restore a more favorable arousal state for tDCS, suggesting that the two techniques are not merely complementary but can be mechanistically interdependent in ways that have not yet been systematically explored.

Therefore, in this review, we will first explore the neurobiological mechanisms of the stress response, focusing on the PFC and its dysregulation under stress. Next, we will assess the current evidence for tDCS and taVNS as tools for regulating stress responses, based on behavioral, physiological, and subjective measures, and their current shortcomings. Finally, we will develop a mechanistic rationale for dual-modality stimulation, discuss relevant empirical findings, and identify key challenges and future directions to scale this approach.

## 2 Neurobiology of stress

### 2.1 Dual pathways: SAM and HPA

When a stimulus is perceived as aversive, uncontrollable, novel, and unpredictable, thereby increasing the allostatic load, it triggers a cascade of evolutionarily conserved, multi-system responses to maintain homeostasis (McEwen and Gianaros, 2011). The amygdala plays a central role in this cascade by integrating inputs from the thalamus, visual system, sensory cortices, and PFC (Arnsten et al., 2015; Phelps and LeDoux, 2005). This leads to activation of two major stress-response pathways: the sympathetic-adrenomedullary (SAM) pathway and the hypothalamic–pituitary–adrenal (HPA) axis (Godoy et al., 2018). In isolated bursts, this stress response is adaptive and necessary for survival, but persistent activation of these pathways leads to chronic stress and becomes an upstream driver for a range of psychological and neurological disorders (Sapolsky, 2004).

The SAM pathway is the initial response to a threat. Its activation results in the release of epinephrine and norepinephrine (NE) from the adrenal medulla, thereby increasing sympathetic nervous system (SNS) activity (De Kloet et al., 2005; Joëls and Baram, 2009). SNS and Parasympathetic Nervous System (PNS) are the two contrasting arms of the Autonomic Nervous System (ANS). Increased SNS activity prepares the body for action by increasing heart rate (HR), increasing respiration, and decreasing digestive processes, among other adaptive changes (Godoy et al., 2018; Russell and Lightman, 2019). At the same time, increased SNS activity reduces PNS activity, which supports calming and restorative functions. This PNS activity is, in turn, primarily mediated by the Vagus Nerve (10th Cranial Nerve), helping to regulate physiological arousal (Aranberri Ruiz, 2024; Ma et al., 2025). Thus, during stress, the body shifts toward a state of heightened physiological arousal due to reduced PNS activity (Godoy et al., 2018).

In parallel, activation of the HPA leads to the release of corticotropin-releasing hormone (CRH) from the hypothalamus, which stimulates downstream pathways to act on the adrenal cortex to produce glucocorticoids, primarily cortisol in humans (De Kloet et al., 2005). This pathway is slower than the SAM system, with cortisol levels typically peaking around 20 min after the onset of the stressor (Becker and Rohleder, 2019). This slow release of cortisol plays a crucial role in sustaining the stress response by mobilizing energy resources and influencing memory and emotional processing, with prolonged HPA activation contributing to structural and functional impairments in PFC function (Herman et al., 2016). In parallel to this slower-acting endocrine pathway, stress also engages neurochemical mechanisms mediated by catecholaminergic systems.

### 2.2 The role of catecholamines and the Locus Coeruleus-norepinephrine system

Amygdala activation, leading to increased Locus Coeruleus (LC) activity, which in turn mediates widespread catecholamine (e.g., NE) release throughout the Central Nervous System (CNS), with a particularly pronounced effect in the PFC (Arnsten, 2011). At moderate levels, NE optimizes alertness and attention through high-affinity postsynaptic α2A adrenoreceptors in the PFC. However, under increased stress, elevated NE levels preferentially activate lower-affinity α1 and *β*-adrenoreceptors, triggering intracellular signaling cascades that weaken recurrent PFC firing and impair task-relevant control (Arnsten, 2011; Cools and D’Esposito, 2011). Consistent activation of LC-NE activity and suppression of PFC activity then leads to structural changes, including dendritic retraction and atrophy in the pyramidal cells of the PFC and the hippocampus, as well as dendritic arborization and hypertrophy in the amygdala (Woo et al., 2021). Thus, it reinforces the amygdala’s vicious pathways, leading to increased reactive behavior and disruption of PFC-controlled goal-directed behavior (Arnsten, 2009; Davis et al., 2017). The impact of increased LC-NE activation is also seen across the limbic system, including the hippocampus, nucleus accumbens (NAc), and anterior cingulate cortex (ACC), impairing memory, reward processing, and inhibitory control (Arnsten et al., 2015). Importantly, LC activity also suppresses vagal output, leading to further physiological changes, such as reduced heart rate variability (HRV), an essential marker of vagal activity (Ma et al., 2025). This LC-driven reduction in vagal tone not only reflects autonomic imbalance but also has important downstream consequences for PFC functioning and cognitive control under stress. Targeting this autonomic imbalance has been explored by the NIBS approach of taVNS, which has been shown to influence LC-NE activity via ascending vagal afferents (Hilz, 2022).

### 2.3 Prefrontal dysregulation and impact on cognition

PFC is the seat of higher-order cognitive functions, including decision-making, social cognition, and emotional regulation (Kolk and Rakic, 2021). Of particular interest are the ventromedial PFC (vmPFC), dorsolateral PFC (dlPFC), and medial PFC (mPFC) subdivisions of the PFC, which have established roles in top-down cognitive control (Kolk and Rakic, 2021; Friedman and Robbins, 2021). PFC also has an abundance of adrenergic receptors (which are bound by NE) and projections from the LC, making it highly susceptible to the detrimental effects of stress due to changes in catecholamine levels (Arnsten, 2009). Thus, chronically stressed and acute stress interventions consistently show impairments in a range of cognitive tasks (Shields et al., 2016; Girotti et al., 2024).

Under safe, non-stressed conditions, the PFC exerts top-down control over behavior by fine-tuning its own catecholaminergic input through direct and indirect projections to the LC. This top-down control allows the PFC to modulate activity in limbic structures, including the amygdala and parts of the basal ganglia, such as the NAc. But, under stressful conditions, amygdala activation increases catecholamine release, which suppresses PFC activity and strengthens amygdala-driven responses. Through projections to the striatum, the amygdala enhances reflexive and habitual behaviors, reducing reliance on the PFC’s slower, more deliberate processing (Arnsten et al., 2015; Arnsten, 2011; Woo et al., 2021; Cardinal et al., 2002).

In this stress response cascade, the amygdala plays a critical role as the primary initiator, particularly in response to emotional stressors. However, the amygdala’s activity is itself regulated by the PFC (Hiser and Koenigs, 2018). Paradoxically, while the PFC is highly vulnerable to stress-induced disruption, under conditions of perceived control, it retains the capacity to suppress the stress response via top-down regulation of the LC-NE system (Meine et al., 2021). This ability of the PFC to exert top-down control over stressors has thus been explored in relation to the NIBS approach of tDCS, in which stimulation over prefrontal areas such as vmPFC, dlPFC, and mPFC has been shown to increase stress regulation and reduce the influence of stressors on cognition (Feeser et al., 2014; Roy et al., 2025; De Smet et al., 2024; Brunoni et al., 2013).

## 3 NIBS for stress regulation: current evidence

### 3.1 Transcranial direct current stimulation

One of the most widely studied forms of NIBS in cognitive and affective neuroscience is tDCS (Woods et al., 2016). It involves applying low-intensity direct current through scalp electrodes to modulate cortical excitability. These currents pass through the skull, dura mater, pia mater, and cerebrospinal fluid to modulate activity in the underlying gray and white matter (Weise et al., 2022; Nitsche et al., 2008; Yavari et al., 2018). The direction of modulation is polarity-dependent: under standard parameters, anodal stimulation (inward current flow) depolarizes the neuronal resting membrane potential, increases spontaneous firing rates and network excitability, while cathodal stimulation exerts the opposite effect (Yavari et al., 2018; Stagg and Nitsche, 2011). Crucially, tDCS exerts both immediate (acute) effects via ion-channel-based receptors and long-term (neuroplastic) changes in synaptic efficacy via glutamatergic receptors dependent on long-term potentiation (LTP) and long-term depression (LTD)-like mechanisms (Stagg and Nitsche, 2011). Importantly, tDCS is a cost-effective, portable, safe, and well-tolerated intervention, with no serious adverse events (SAE’s) reported across hundreds of studies. However, mild and transient side-effects, including scalp tingling, itching, headache, and skin redness at the electrode site, are commonly reported (Woods et al., 2016; Bikson et al., 2016).

Prefrontal tDCS has shown effects on subjective, physiological, and behavioral markers of stress regulation, though findings remain inconsistent (Hart-Pomerantz et al., 2024; Smits et al., 2020). The majority of studies with anodal stimulation over the medial and dorsolateral PFC report reduced negative affect and state anxiety following stress exposure (Feeser et al., 2014; Gao et al., 2024; Abend et al., 2019; Wang et al., 2022; dos Reis et al., 2024; Brunelin and Fecteau, 2021; Liu et al., 2023; Marques et al., 2018). Similarly, in terms of physiology, prefrontal tDCS has been shown to reduce HR (Coelho et al., 2025; Wang et al., 2023; Carnevali et al., 2020), modulate cardiac parameters such as HRV (Brunoni et al., 2013; Coelho et al., 2025; Carnevali et al., 2020), blunt cortisol (Roy et al., 2025; Brunoni et al., 2013; Brunelin and Fecteau, 2021; Carnevali et al., 2020; Antal et al., 2014), modulate pupil size (Gao et al., 2024), and decrease skin conductance (Feeser et al., 2014) with mixed meta-analysis reports on its efficacy for stress regulation (Hart-Pomerantz et al., 2024; Smits et al., 2020; Vignaud et al., 2023). Timings also matter, with stimulation delivered before or during stress exposure typically producing stronger effects than post-stress application (Vignaud et al., 2023). Prefrontal tDCS has also been shown to modulate behavioral performance generally impaired by stress, such as working memory (Roy et al., 2025; De Smet et al., 2024; Bogdanov and Schwabe, 2016), attention (Liu et al., 2023; Ironside et al., 2019), and creativity (Wang et al., 2022). Additionally, prefrontal stimulation has been shown to reduce amygdala threat reactivity in individuals with high trait anxiety, suggesting that prefrontal tDCS can influence subcortical stress circuitry beyond the cortex itself (Ironside et al., 2019). However, although the majority of studies reported positive effects of tDCS on stress regulation, findings remain mixed, as shown by a meta-analysis in which only 44% of studies reported stress-mitigating physiological effects. In comparison, 38% found no significant change, highlighting substantial variability across studies (Hart-Pomerantz et al., 2024). This inconsistency likely reflects differences in montage (unipolar vs. multipolar), stimulation parameters (current density, duration), timing relative to stressor onset, individual-level factors (such as baseline arousal, trait anxiety, scalp-to-cortex distance), stress induction paradigms (video paradigms, TSST), and sample characteristics (blinding, placebo-control; Bremner et al., 2020; Woods et al., 2016; Smits et al., 2020; Vignaud et al., 2023; Vergallito et al., 2022). Underscoring the need for more rigorous, standardized approaches to reduce variability and enhance the reliability of tDCS effects on stress regulation.

### 3.2 Trans auricular vagus nerve stimulation

One complementary non-invasive neuromodulation approach is taVNS, which targets the vagus nerve via cutaneous stimulation of the ear (especially in the concha region). The Vagus Nerve is a major component of the PNS and plays a vital role in autonomic regulation. It promotes “rest and digest” functions and induces a calming effect following the cessation of the acute stress response (Hilz, 2022; Aranberri Ruiz, 2024). Its ascending afferent fibers project to the nucleus of the tractus solitarius (NTS), the primary relay point for vagal nerve fibers to the brain, which in turn modulates activity in the LC, amygdala, NAc, and PFC, providing a direct anatomical substrate through which peripheral autonomic signals can influence central, emotional, and cognitive processing (Bremner et al., 2020; Ma et al., 2025). While invasive vagus nerve stimulation is an approved treatment for conditions like epilepsy, depression, and chronic pain, its surgical nature poses risks and side effects (Hilz, 2022). In contrast, taVNS offers a non-invasive, portable, and easy alternative, with no SAEs reported. However, mild side-effects, including skin irritation at the electrode site, tingling, and occasional headache, have been reported (Hilz, 2022; Kim et al., 2022).

Evidence for taVNS effects on stress regulation has been explored across a range of physiological domains. The most consistent findings of taVNS are improvements in autonomic responses to stress, including increased vagal activity (Bremner et al., 2020; Wang et al., 2023; Carnevali et al., 2020) and blunted cortisol response (Cuberos Paredes et al., 2025; Warren et al., 2019), with mixed findings for psychophysiological markers of stress regulation (Bremner et al., 2020). Indirect measures of LC activity and NE levels, such as HRV, pupillary responses, and fMRI-related signals, indicate that taVNS can potentially regulate this system (Zhi et al., 2024; Zaehle et al., 2021). Additionally, taVNS stimulation during or before a stressor leads to parasympathetic activation, reflecting increased autonomic regulation (Drost et al., 2025). Subjectively, taVNS has been shown to reduce negative effects and reduce perceived stress (dos Reis et al., 2024; Zhao et al., 2025). EEG and MRI findings also suggest broader network-level changes in cortical and subcortical activation, as well as activation in NTS and brainstem regions, thereby modulating bottom-up pathways (Bremner et al., 2020; Molaeizadeh et al., 2026; Hilz, 2022; Zhi et al., 2024; Badran et al., 2018; Gianlorenco et al., 2022). At the behavioral level, a meta-analytic review supports taVNS-related improvements in cognitive performance, including attention and memory, with some studies reporting mixed findings on cognitive outcomes (Ridgewell et al., 2021; Borges et al., 2020). As with tDCS, the effects of taVNS also vary considerably based on stimulation parameters (site of stimulation, duration, pulse width, frequency), individual-level parameters (baseline arousal, resting HRV), timing of stimulation, sample characteristics, among other factors (Hilz, 2022; Kim et al., 2022; Thompson et al., 2021). Additionally, a meta-review noted that taVNS temporarily increases PNS activity, but the effects are short-lived (Benzouak et al., 2025), further underscoring the need for approaches that can stabilize these effects.

## 4 Toward a combined approach: tDCS and taVNS as a dual-modality intervention

**Figure 1:**
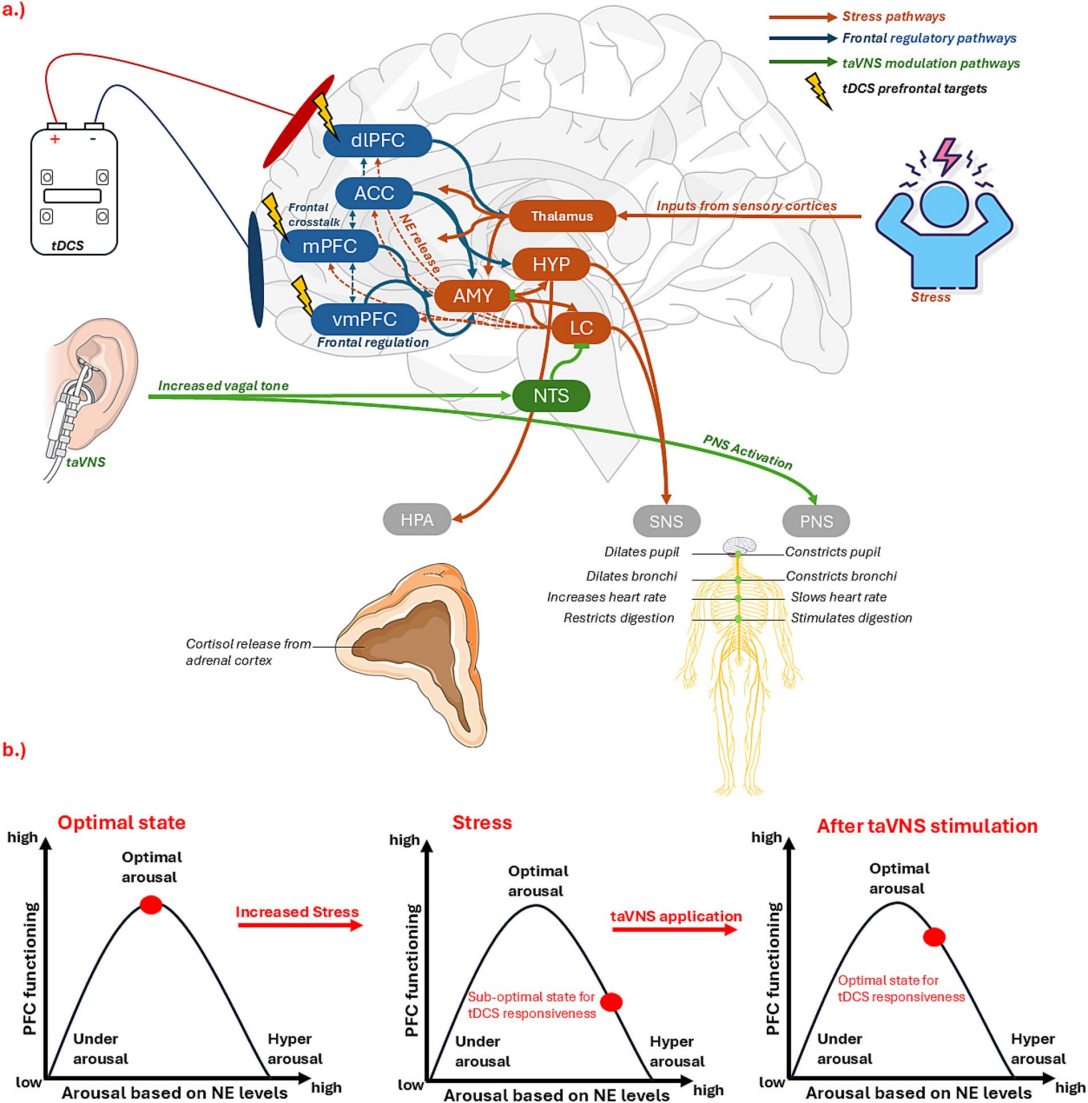
Prospective mechanism behind the combined stimulation approach. **(a)** When a stimulus is perceived as aversive and stressful (as shown by a blue stressed man), it is processed in the sensory cortices and, via the thalamus, relayed to the amygdala. An increase in the activity of the amygdala leads to activation of the two key stress pathways: the HPA axis (mediated by the hypothalamus), leading to cortisol release from the adrenal cortex, and the SNS pathway (and the LC-NE system), leading to an increase in levels of catecholamines in the brain (shown as dotted dark orange line) and in the body. These pathways form the classic stress response, and a representation of the pathway is shown in dark orange. In parallel, the thalamus also transmits information to the PFC, where the stimulus is evaluated based on prior experience. If the individual has previously perceived control over similar stimuli, the PFC exerts top-down inhibitory control over the amygdala, modulating emotional responses. This regulatory influence involves communication among the PFC subregions, including the ACC, vmPFC, dlPFC, and mPFC. However, during acute stress, this prefrontal regulation is often diminished due to stress-induced weakening of PFC activity. This regulatory pathway is shown in blue. Concurrent stimulation with prefrontal tDCS and taVNS can have a possible synergistic effect on these pathways, leading to a more robust reduction in the stress response. External stimulation of the PFC can strengthen connectivity among other PFC regions, improving emotional regulation and enhancing control over the stressor. Since the PFC can exert inhibitory control over the amygdala and limbic regions, prefrontal tDCS stimulation may suppress stress responses, ultimately reduce the perceived impact of the stressor, and enhance recovery. Possible targets of prefrontal tDCS are shown with a current sign that can be reached by scalp tDCS. Additionally, external stimulation of the auricular branch of the vagus nerve would enhance parasympathetic activation (increased vagal tone), directly counteracting sympathetic activation. This would lead to increased activity in the NTS, with its extensive projections to the LC-NE system, which would then influence activity in the amygdala and the HPA axis, reducing the stress response and thus providing bottom-up regulation of downstream stress pathways. This pathway is shown in green. Thus, a combined stimulation approach would lead to reciprocal regulation of the stress response by targeting both top-down and bottom-up processes, resulting in a possible sustained and increased cognitive control and a reduced physiological impact of stressors on the body. Areas marked on the brain are only for representational purposes and do not show the exact location or size of the area. LC, Locus Coeruleus; ACC, anterior cingulate cortex; mPFC, medial Prefrontal cortex; HYP, Hypothalamus; vmPFC, ventromedial prefrontal cortex; dlPFC, dorsolateral prefrontal cortex; NTS, Nucleus Tractus Solitarius; tDCS, transcranial direct current stimulation; taVNS, transauricular vagal nerve stimulation; HPA, Hypothalamus-Pituitary Adrenal axis; SNS, sympathetic nervous system; PNS, parasympathetic nervous system. **(b)** Plot showing the U-shaped dose–response relationship between arousal (based on NE levels), shown on the x-axis, and PFC functioning, shown on the y-axis. In an optimal state (indicated by a red circle on the U-curve), with optimal arousal, PFC functioning is at its peak and performance is high, whereas under stress-induced NE release, excessive NE leads to hyperarousal and disruption of PFC functioning. Under the combined stimulation approach, we theorize that taVNS application might induce a leftward shift on this graph (shown with a red circle on the U-curve) by reducing arousal, and since the effects of tDCS are arousal-dependent, it would lead to an optimal state of tDCS responsiveness.

Conceptually, both techniques target functionally distinct yet converging pathways for stress regulation. We refer to Figure 1 to illustrate the mechanism behind the combined stimulation approach. Prefrontal tDCS can modulate the top-down control of the PFC over downstream areas, such as the limbic system, as shown in a range of studies (Feeser et al., 2014; Roy et al., 2025; Abend et al., 2019; Brunoni and Vanderhasselt, 2014). Stress increases arousal, which is known to exhibit an inverted-U relationship with PFC functioning (Arnsten, 2011), where too high or too low arousal impairs performance; similarly, the effect of tDCS also seems to follow this relationship. Empirical evidence shows a state-dependent effect of tDCS, where moderate arousal enhances its effects (Allaert et al., 2026; Esposito et al., 2022; Clarke et al., 2020), whereas hyperarousal impairs them, especially under intense stressors and in mental disorders associated with increased arousal (Aksu et al., 2022; Frase et al., 2019; de Lima et al., 2019; Miler et al., 2026; Smits et al., 2021; Estaji and Ahmadizadeh, 2026; Stein et al., 2020). This is where taVNS stimulation comes into play, which can possibly modulate arousal and the stress response by targeting bottom-up autonomic centers in the brain, such as the NTS, LC-NE, and the limbic system, as inferred from indirect physiological measures (Bremner et al., 2020; Zhi et al., 2024; Zaehle et al., 2021). This ascending vagal modulation could dampen autonomic hyperarousal and reduce the gain of stress-responsive neural circuits, thereby inducing a leftward shift in the inverted-U response curve and making the cortex more receptive to regulatory input from anodal PFC stimulation (see Figure 1).

Mechanistically, this brain-state dependence can be understood at the cellular, systems, and network levels. At the cellular receptor level, tDCS-induced neuroplasticity is thought to depend on NMDA receptor-mediated long-term potentiation (LTP)-like mechanisms, which are activity-dependent and therefore highly sensitive to prevailing membrane polarization states (Stagg and Nitsche, 2011; Kronberg et al., 2020). Elevated NE release under stress activates lower-affinity α1 and *β*-adrenoreceptors, triggering intracellular cascades (including PKA and PKC signaling) that can destabilize persistent cortical firing and raise the threshold for LTP induction (Arnsten, 2011). By dampening this NE level-based arousal, as evidenced indirectly by taVNS-associated changes in physiology, taVNS may help restore the membrane polarization state and receptor occupancy profile under which anodal tDCS can more reliably shift neurons toward LTP-like plasticity. At the systems level, stress-related hyperarousal is associated with shifts in the cortical excitatory-inhibitory (E/I) balance, with reduced GABAergic inhibition and excess glutamatergic drive disrupting the fine-tuned E/I ratio necessary for effective cortical modulation (Yavari et al., 2018). Vagal afferent stimulation has been linked to changes in cortical oscillatory synchrony, particularly in the alpha and theta bands, reflecting coordinated GABAergic and glutamatergic network dynamics (Molaeizadeh et al., 2026; Gianlorenco et al., 2022). Since tDCS effects are also oscillation-dependent (Esposito et al., 2022), taVNS-driven normalization of oscillatory state may represent an additional mechanism through which bottom-up vagal input shapes the cortical response to subsequent anodal stimulation. At the network level, NE levels also gate the signal-to-noise ratio in PFC networks, depending on their level (Arnsten, 2011): in the optimal arousal zone, NE, via α2A receptors, strengthens task-relevant representations, whereas excess NE degrades this signal. As taVNS could modulate ascending noradrenergic tone, it may restore this gating function and thereby make PFC circuits more selectively responsive to the excitability increase induced by anodal tDCS. Taken together, these mechanistic layers at cellular, systems, and network levels provide converging neurophysiological rationale for why brain state, as shaped by taVNS, could influence tDCS responsiveness, highlighting a clear direction for future empirical validation. This brain-state dependence is consistent with activity-dependent plasticity and priming effects of tDCS, in line with Hebbian principles, whereby previously primed/activated circuits (e.g., via taVNS) exhibit stronger and more enduring responses to subsequent stimulation compared to stimulation in isolation (Kronberg et al., 2020; Abbott and Nelson, 2000). In practical terms, this suggests that taVNS should ideally precede or accompany tDCS to reduce hyperarousal before cortical modulation is applied.

Thus, reductions in arousal via taVNS targeting vagal afferents and the LC-NE system can lead to reduced NE levels in the PFC, as inferred from previous studies. This putative reduction in NE levels may thereby facilitate a greater regulatory effect of prefrontal tDCS. Since tDCS is known to be arousal-dependent, a possible PFC state with moderate arousal would enhance the effects of prefrontal tDCS. In turn, strengthened PFC regulatory output could enhance modulation of downstream limbic targets, forming a positive regulatory loop that supports more efficient stress regulation. Since these two techniques target complementary regulatory pathways: top–down cortical control via anodal PFC stimulation and bottom–up autonomic modulation via vagal afferents, when used together, they may produce synergistic effects, though antagonistic interactions also remain plausible (see section 6).

A prior conceptual framework proposed combining bottom-up vagal modulation via resonant breathing with top-down prefrontal control via prefrontal tDCS (Vanderhasselt and Ottaviani, 2022), but no compelling empirical evidence was reported (Li et al., 2024). We thus further refine this framework by proposing the use of taVNS bottom-up modulation efficacy to couple top-down control induced by tDCS effectively. Our proposed framework is also further supported by a recent feasibility experiment showing that simultaneous prefrontal tDCS and taVNS is possible, well-tolerated, and produces more widespread brain activation in limbic and prefrontal regions than either technique alone, as measured by functional MRI (Sun et al., 2021). Exploration of this framework for stress regulation remains uncharted territory. A few empirical studies on the combined application of tDCS and taVNS for cognitive tasks have yielded encouraging findings, in which combined left dlPFC anodal stimulation with taVNS led to higher WM performance than either modality alone (Zhao et al., 2022). While another study using anodal stimulation over the M1 motor cortex in combination with taVNS also reported superior benefits for gait and balance parameters in subacute stroke patients compared with either modality alone or sham (Wang et al., 2024).

## 5 Aims, challenges, and future directions

The first major aim remains to explore whether combined tDCS and taVNS produces synergistic effects for stress regulation. A double-blind, crossover, sham-controlled design comparing the effects of combined stimulation with each stimulation modality alone and with sham for stress regulation is a logical way forward, but to date has not been investigated. To note, an antagonistic effect is also mechanistically plausible: if taVNS over-suppresses arousal, it could shift the system below the optimal zone of the inverted-U, thereby reducing rather than enhancing tDCS-driven neuroplasticity. Similarly, both techniques engage overlapping subcortical circuits (e.g., the amygdala and LC) via distinct afferent pathways, raising the possibility of competitive rather than cooperative modulation. However, existing positive findings from combined tDCS and taVNS protocols (Sun et al., 2021; Zhao et al., 2022) suggest that synergistic effects are more likely. To further empirically confirm this, these first studies should be complemented by neuroimaging inferences (including electric field modeling) and psychophysiological readouts.

The further major aim in the case of a synergistic effect should be to determine the optimal timing of stimulation relative to stress exposure. According to the principle of brain-state dependence and previous meta-analyses, delivering stimulation during active stress is theoretically and empirically ideal because it yields the greatest benefits (Hart-Pomerantz et al., 2024; Ironside et al., 2019) and may enhance neuroplasticity by engaging amygdala–PFC circuits at their most active state. The next aim in a similar study would be to understand whether both modalities should be applied simultaneously or with a slight delay, for example, whether taVNS should be given before tDCS to prime circuits, or both modalities should be applied simultaneously, which presents a vast area for systematic research. Additionally, later empirical studies should explore the ideal duration, intensity, and dose–response relationship of combined protocols, using blinded, crossover, and sham-controlled designs.

The major challenge for most NIBS techniques is the substantial variability among individuals, which reduces statistical power and complicates clinical translation. For tDCS, this variability arises from anatomical differences in skull shape and the scalp-to-cortex distance, which affect the distribution of the electric field (Woods et al., 2016; Guerra et al., 2020). Additionally, biological factors, including trait anxiety, HPA reactivity, and baseline neurophysiology, also influence outcomes (Hilz, 2022; Nitsche et al., 2008; Vergallito et al., 2022; Guerra et al., 2020). But recent advances in addressing these limitations have been made for both tDCS and taVNS, driven by significant strides in the development of individualized stimulation to reduce this variability (Rektorová et al., 2025).

A key future direction for this framework would be clinical translation. Populations with depression, anxiety, and PTSD, conditions fundamentally characterized by impaired stress regulation, share the precise neurobiological profile targeted by this approach, such as hypoactive PFC, hyperreactive amygdala, and reduced vagal tone, suggesting a potentially wider therapeutic window (Jiang et al., 2024). Finally, the portability and wearability of both tDCS and taVNS offer practical advantages over TMS or MRI-based methods and may support future real-world applications (Badran et al., 2022; Bikson et al., 2026). However, substantial barriers remain before widespread deployment becomes feasible. These include optimization of individualized stimulation parameters, long-term safety monitoring, adherence, regulatory approval, and validation of efficacy outside controlled laboratory settings.

## 6 Conclusion

Stress places the brain in a state of heightened arousal that weakens prefrontal control and amplifies amygdala-driven responses. The existing evidence for both prefrontal tDCS and taVNS as individual tools for stress regulation is promising but remains mixed, with effects that vary across individuals and are transient over time. Crucially, these two limitations may be mutually addressable: taVNS, by dampening autonomic hyperarousal and normalizing LC-NE-mediated PFC suppression, could create a more favorable brain state in which prefrontal tDCS exerts its greatest neuroplastic effect. This mechanistic complementarity provides a theoretical basis for a dual-modality approach that could engage complementary regulatory loops that may be difficult to engage simultaneously with either technique alone. However, this proposition requires empirical validation. Thus, empirical evidence from controlled mechanistic studies is needed to substantiate this novel framework for stress regulation.
